# Intra-arterial nimodipine and outcomes in delayed cerebral ischemia after aneurysmal subarachnoid hemorrhage

**DOI:** 10.1016/j.bas.2026.106281

**Published:** 2026-08-10

**Authors:** Emilia K. Pesonen, Sonja Portin, Tommi K. Korhonen, Juha-Matti Isokangas, Mikael von und zu Fraunberg, Cheng Qian

**Affiliations:** a Department of Neurosurgery, Oulu University Hospital & University of Oulu, Oulu, Finland; b Department of Radiology, Oulu University Hospital, Oulu, Finland

**Keywords:** Intra-arterial nimodipine, Aneurysmal subarachnoid hemorrhage, Cerebral vasospasm, Delayed cerebral ischemia

## Abstract

**Introduction:**

Vasospasm after aneurysmal subarachnoid hemorrhage (aSAH) may cause delayed cerebral ischemia (DCI). Intra-arterial nimodipine (IAN) is used in vasospasms resistant to standard hemodynamic therapy. The effect of IAN on long-term outcomes remains uncertain, particularly in patients requiring repeated infusions.

**Research question:**

What are the outcomes of aSAH patients treated with IAN, especially compared to the characteristics of patients undergoing repeated versus single IAN infusions.

**Material and methods:**

We retrospectively reviewed all aSAH patients with DCI treated with IAN at a tertiary referral center between 2017 and 2024. Logistic regression was used to identify predictors of unfavorable outcome (modified Rankin Scale scores >2) at 3–6 months.

**Results:**

Fifty-two patients (mean age 53 years, 65% female) were included. At 3–6 months, 28 patients (54%) had unfavorable outcomes. After propensity score matching for baseline severity of SAH, repeated IAN was associated with unfavorable outcome compared to single IAN (OR 5.13, 95% CI 1.53–19.35) using Firth's penalized logistic regression. Seven patients (13%) died during the six-month follow-up. Mortality did not differ significantly between single and repeated IAN infusion groups (OR 0.74, 95% CI 0.15–3.44).

**Discussion and conclusions:**

In this cohort of aSAH patients with vasospasm-related DCI treated with IAN, repeated IAN was associated with unfavorable outcomes. However, mortality did not differ significantly between single and repeated IAN groups. Earlier treatment was not associated with improved outcomes.

## Highlights

• Clinical data on intra-arterial nimodipine for vasospasm remain limited.
• Repeated nimodipine infusions linked to worse outcomes than single doses.
• Outcomes not superior to historical DCI cohorts without intra-arterial therapy.

## Abbreviations and acronyms:

aSAH = : aneurysmal subarachnoid hemorrhage
CI = : confidence interval
DCI = : delayed cerebral ischemia
EVD = : external ventricular drainage
GCS = : Glasgow Coma Scale
H&H = : Hunt and Hess
IAN = : intra-arterial nimodipine
ICU = : intensive care unit
IVH = : intraventricular hemorrhage
IQR = : interquartile range
MCA = : middle cerebral artery
mg = : milligram
mRS = : modified Rankin Scale
OR = : odds ratio
SD = : standard deviation

## 1 Introduction

Cerebral vasospasm after aneurysmal subarachnoid hemorrhage (aSAH) is a major cause of delayed cerebral ischemia (DCI), which adversely affects outcomes if not managed effectively (Bashir et al., 2016). Current practice favors euvolemia and induced hypertension as first line treatments over the previously utilized Triple-H therapy (Solou et al., 2023). However, a subset of patients develops refractory DCI that does not respond to these conservative measures (Velat et al., 2011; Washington and Zipfel, 2011). In such cases, endovascular rescue strategies, including intra-arterial nimodipine (IAN), have been accepted as standard practice (Picetti et al., 2024).

International surveys indicate that intra-arterial procedures are widely used as second-line therapy for DCI, with nimodipine as the most commonly administered vasodilator (Picetti et al., 2024). IAN has been associated with radiological vasodilation, increased cerebral perfusion and temporary clinical benefit in patients with symptomatic vasospasm (Biondi et al., 2004; Hänggi et al., 2008). However, repeated infusions may be required, complications are possible and evidence on which patients may benefit from such treatment remains limited (Kim et al., 2015; Samuelsson et al., 2022; Steiner et al., 2013). The optimal timing of IAN is also unclear, though some studies suggest early intervention may be advantageous compared to a delayed approach (Bashir et al., 2016; Jabbarli et al., 2019; Samuelsson et al., 2022).

In the current study, we evaluated the factors affecting outcomes of patients with post-aSAH DCI who were treated with IAN and examined the characteristics of patients who required multiple administrations of IAN. We also assessed whether the timing of IAN was associated with mortality or unfavorable functional outcomes.

## 2 Methods and materials

### 2.1 Study population

**Fig. 1:**
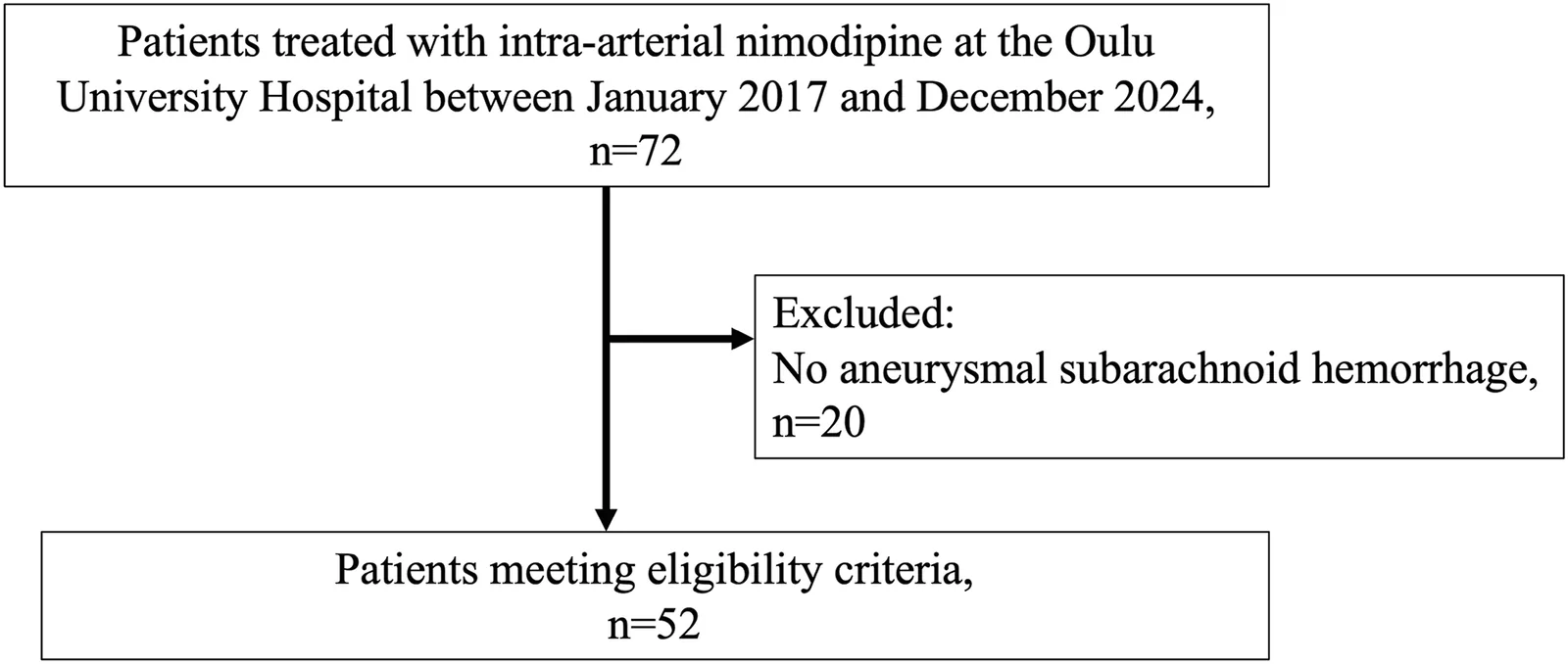
Flowchart of patient selection.

We retrospectively identified all patients with aSAH who received IAN for DCI at Oulu University Hospital between January 2017 and December 2024. Cases were retrieved from the electronic patient records using a national radiological procedure code for IAN administration. Patients with non-aneurysmal subarachnoid hemorrhage were excluded (Fig. 1).

Demographic and clinical variables were collected. Sex refers to the classification of patients as male or female based on sex assigned at birth. Clinical and radiological severity were assessed using the Hunt and Hess (H&H) scale and Fisher grade from the admission CT scan, respectively (Fisher et al., 1980; Hunt and Hess, 1968). Functional outcome was evaluated using the modified Rankin Scale (mRS) at 3–6 months after discharge. Patients were classified as smokers if active smoking was mentioned in their records.

Post-aneurysm treatment ischemic lesion was defined as a new ischemic lesion corresponding to a vascular distribution on CT performed one day after aneurysm treatment. Post-IAN treatment ischemic lesion was defined as a new hypodense area of the certain vascular territory occurring despite IAN “Overall ischemic lesion” refers to the finding of post-aneurysm and/or post-IAN treatment ischemic lesions.

The study was approved by the institutional review board of Oulu University Hospital (permit number K62719) and conducted in accordance with the Declaration of Helsinki. Informed consent was waived because patients were not contacted in the study.

### 2.2 Management of delayed cerebral ischemia

At our institution, ruptured aneurysms are primarily treated endovascularly within 24 h of admission. Microsurgical clipping was preferred for aneurysms of the middle cerebral artery (MCA) or when a concurrent space-occupying intraparenchymal hematoma was present.

DCI was suspected when a new focal neurological deficit or a ≥2-point decrease in Glasgow Coma Scale (GCS) was observed in conjunction with radiological vasospasm (Vergouwen et al., 2010). CT angiography, with or without perfusion sequences, was used for the diagnosis of radiological vasospasm and/or hypoperfusion. CT perfusion parameters were analyzed with RapidAI software by a radiologist. Infarct core was defined as mean cerebral blood flow <30%, and tissue at risk as time-to-maximum >6 s. Patients with DCI received intensified therapy with euvolemia and induced hypertension in the intensive care unit (ICU) for 3–4 h. Management followed the American Heart Association/American Stroke Association guidelines which strongly recommend euvolumia, and support induced hypertension in select patients (Hoh et al., 2023). IAN was administered within 24 h of deterioration if the anatomical location of the vasospasm corresponded to the neurological deficit and the period of euvolemia and induced hypertension did not result in clinical improvement in case of a new focal neurological deficit or a ≥2-point decrease in GCS. The number or territory of vessels (left/right internal carotid artery, posterior circulation) with significant vasospasm was recorded and graded on a scale of 1–3. Stenosis greater than 50% was considered significant.

**Fig. 2:**
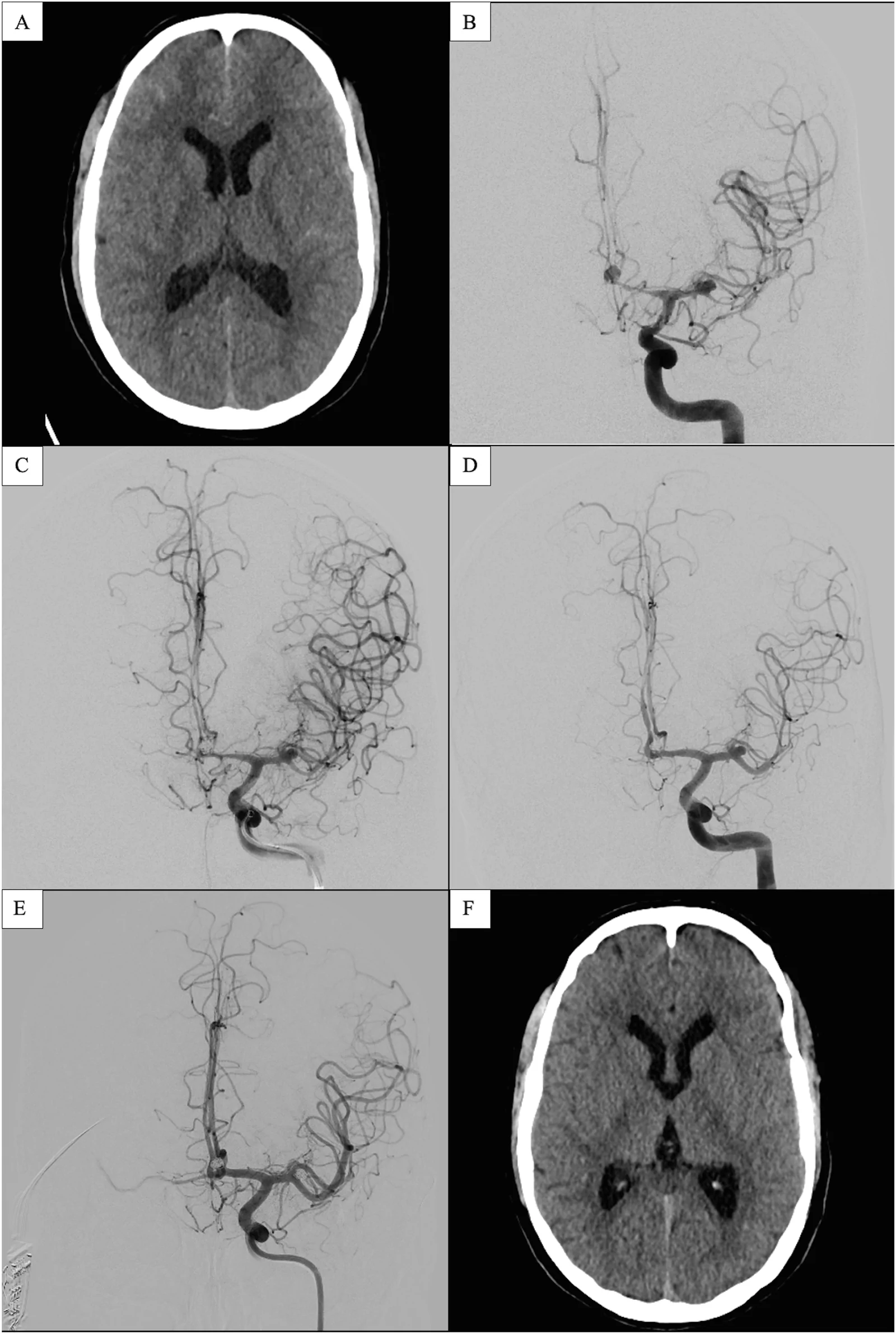
Computed tomography (CT) and digital subtraction angiography (DSA) images of a 44-year-old male, who had an acute onset of headache 9 days before the admission. His headache was initially clinically diagnosed as migraine, but he later presented to the emergency department due to acute-onset hemiparesis. **1A**: A CT head scan with hyperdensity in cortical sulci suggesting subarachnoid hemorrhage. **1B**: In the immediate DSA, intracranial aneurysms in the anterior communicating artery (ACoA) and left middle cerebral artery bifurcation were found concurrently with severe vasospasm of both anterior and middle cerebral arteries. **1C**: The ACoA aneurysm was considered ruptured, and it was endovascularly coiled immediately after the diagnostic DSA. Intra-arterial nimodipine (IAN) was administered during the treatment and the vasospasm slightly decreased at the end of the procedure. IAN was administered during three consecutive days. Mechanical angioplasty was also conducted due to the patient's serious symptoms and severe vasospasm. **1D**: After the third IAN administration, the spasm had significantly decreased. **1E**: The MCA aneurysm was coiled later and vasospasm is no longer visible. **1F**: No ischemic lesions were found on the post-treatment CT scan. Clinically, the patient made an excellent recovery with a 6-month mRS of 0.

After diagnostic angiography, nimodipine was administered into the proximal internal carotid or vertebral artery depending on the target vessel (Fig. 2). A maximum of 2 mg nimodipine per vessel was given, with a maximum dose of 5 mg per session. One to three vessels were treated in a single procedure. Nimodipine was manually infused over 10 min under close blood pressure monitoring. Most procedures were performed with patients awake under conscious sedation. The immediate angiographic results after IAN were categorized as “none/mild” or “significant.”

Adjunctive angioplasty was performed for severe symptomatic vasospasm when deemed anatomically safe, using a balloon (HyperGlide, Medtronic, USA), stent retriever (Trevo, Stryker, Ireland), or Comaneci device (Rapid Medical, Israel). Repeated IAN infusions were performed the next day in cases with recurrent or persistent radiological vasospasm associated with new, recurring or persisting neurological deficits. Hydrocephalus was managed with external ventricular drainage (EVD). In cases of chronic hydrocephalus, ventriculoperitoneal shunts were implanted.

### 2.3 Statistical analysis

The primary outcome was functional status measured using mRS at 3–6 months. Outcomes were dichotomized as favorable (mRS 0–2) or unfavorable (mRS >2). Secondary outcomes were six-month mortality and the development of post-treatment and/or post-IAN ischemic lesions.

A multivariable logistic regression model was constructed using pre-defined clinically relevant covariates: age, H&H grade, Fisher grade, treatment modality, and the admission GCS score. The effects of IAN timing, repeated IAN infusions, EVD, and ventriculoperitoneal shunting on outcomes were assessed. Propensity score 1:1 nearest-neighbor matching was used to balance baseline characteristics (age, H&H grade, Fisher grade, treatment modality, and the admission GCS score) of patients who received a single dose of IAN and those that received repeated IAN. Outcomes of the matched dataset were compared using Firth's penalized logistic regression. Continuous variables were reported as means with standard deviations (SDs) or medians with interquartile ranges (IQRs) as appropriate. Statistical analyses were conducted using IBM SPSS Statistics for Windows version 24.0 (IBM Corp., Armonk, NY, USA) and R version 4.5.3 (R Foundation for Statistical Computing, Vienna, Austria). A two-sided p-value <0.05 was considered statistically significant.

## 3 Results

### 3.1 Study population

Fifty-two patients with a mean age of 53 years (SD 13), of whom 34/52 [65%] were females, were included in the study (Table 1). A median of 1.0 (range from 1 to 5) IAN infusions was administered per patient. The GCS scores, recorded at the time of IAN treatment, when compared GCS scores after 6 h, improved in 22/52 (42%) patients after IAN: the scores increased by two or more points in 13 (25%) patients and by one point in 9 (17%) patients. The GCS scores remained the same in 12 (23%) patients and decreased in 18 (35%) patients. Angioplasty was administered as an adjunct therapy to IAN in 4/52 (8%) patients, when there was a significantly stenosed segment in a proximal artery. The angioplasty group had a mean age of 39 years (SD 19).

**Table 1:** Baseline characteristics of patients treated with intra-arterial nimodipine due to delayed cerebral ischemia after aneurysmal subarachnoid hemorrhage.

Characteristic	All patients (n = 52)
Hunt and Hess grade, n (%)	
1–3	32 (61.5)
4–5	20 (38.5)
Fisher grade, n (%)	
0–3	11 (21.2)
4	41 (78.8)
Aneurysm location, n (%)	
Anterior circulation	44 (84.6)
Posterior circulation	8 (15.4)
Number of vessels with significant spams, n (%)	
1	24 (46.2)
2	21 (40.4)
≥3	7 (13.5)
Spasm location, n (%)	
Left anterior circulation	13 (25.0)
Right anterior circulation	10 (19.2)
Posterior circulation	3 (5.8)
Multiple locations	26 (50.0)
Aneurysm treatment, n (%)	
Surgery	17 (32.7)
Endovascular	35 (67.3)
GCS on admission, n (%)	
13–15	33 (63.5)
9–12	5 (9.6)
3–8	14 (26.9)
Presence of ICH, n (%)	
Yes	27 (51.9)
No	25 (48.1)
Presence of IVH, n (%)	
Yes	34 (65.4)
No	18 (34.6)
History of hypertension, n (%)	
Yes	23 (44.2)
No	29 (55.8)
Active smoking, n (%)	
Yes	20 (38.5)
No	32 (61.5)
Anticoagulant and/or antiplatelet therapy, n (%)	
Yes	9 (17.3)
No	43 (82.7)
Timing of nimodipine from initial CT, n (%)	
Early (<7 days)	18 (34.6)
Late (≥7 days)	34 (65.4)
Angiographic improvement after IAN, n (%)	
Significant	15 (28.8)
No/mild	37 (71.2)
Repeated nimodipine, n (%)	
Yes	23 (44.2)
No	29 (55.8)
Angioplasty, n (%)	
Yes	4 (7.7)
No	48 (92.3)
Post-aneurysm treatment ischemic lesion, n (%)	
Yes	14 (26.9)
No	38 (73.1)
Post-IAN treatment ischemic lesion, n (%)	
Yes	8 (15.4)
No	44 (84.6)
Overall ischemic lesion, n (%)	
Yes	21 (40.4)
No	31 (59.6)
Outcome according to mRS, n (%)	
Favorable (0–2)	24(46.2)
Unfavorable (3–6)	28 (53.8)

**ABBREVIATIONS** DCI = delayed cerebral ischemia; GCS = Glasgow Coma Scale; IAN = intra-arterial nimodipine; ICH = intracerebral hemorrhage; IVH = intraventricular hemorrhage; mRS = Modified Rankin Scale; n = number of patients; SD = standard deviation.

### 3.2 Outcomes after intra-arterial nimodipine administration

Seven (13%) of the 52 patients died during the six-month follow-up. Older age and anticoagulant and/or antiplatelet therapy were associated with increased six-month mortality in the univariate models (Table A). Twenty-eight of the 52 patients (54%) had an unfavorable outcome (mRS score >2) at 3–6 months after discharge. Higher Hunt and Hess grade, higher Fisher grade, lower GCS, intraventricular hemorrhage (IVH) and repeated IAN administrations were associated with unfavorable outcomes in univariate analyses (Table 2). Twelve of 23 (52%) patients who received repeated IAN had mRS score ≤ 3 after 3–6 months, compared with 23/29 (79%) who received single-dose only. Overall, 35/52 (67%) patients had mRs score ≤ 3.

**Table 2:** Factors affecting outcomes of patients treated with intra-arterial nimodipine due to delayed cerebral ischemia after aneurysmal subarachnoid hemorrhage. An unfavorable outcome was defined as mRS>2.

Characteristic	Functional outcomes at 3–6 months		
	Favorable (n = 24) (Reference)	Unfavorable (n = 28)	OR (95% CI)
Mean age (SD)	52.5 (12.7)	53.0 (13.1)	1.00 (0.96–1.05)
Sex n (%)			0.39 (0.12–1.27)
Female	13 (38.2)	21 (61.8)	Reference
Male	11 (61.1)	7 (38.9)	
Hunt and Hess grade, n (%)			
1–3	19 (59.4)	13 (40.6)	Reference
4–5	5 (25.0)	15 (75.0)	**4.39 (1.28–15.06)**
Median Fisher grade (IQR)	4 (3–4)	4 (4–4)	**7.25 (1.42–36.94)**
Aneurysm location, n (%)			
Anterior circulation	21 (47.7)	23 (52.3)	Reference
Posterior circulation	3 (37.5)	5 (62.5)	1.52 (0.32–7.16)
Number of vessels with significant spams, n (%)			
1	10 (41.7)	14 (58.3)	Reference
2	11 (52.4)	10 (47.6)	0.65 (0.20–2.11)
≥3	3 (42.9)	4 (57.1)	0.95 (0.17–5.23)
Spasm location, n (%)			
Left anterior circulation	5 (38.5)	8 (61.5)	Reference
Right anterior circulation	4 (40.0)	6 (60.0)	0.94 (0.17–5.07)
Posterior circulation	3 (100.0)	0 (0.0)	N/A
Multiple locations	12 (46.2)	14 (53.8)	0.73 (0.97–2.83)
Treatment, n (%)			
Surgery	8 (47.1)	9 (52.9)	0.95 (0.30–3.03)
Endovascular	16 (45.7)	19 (54.3)	Reference
Median GCS on admission (IQR)	15 (13.8–15)	12 (4.8–15)	**0.82 (0.70–0.95)**
Presence of ICH, n (%)			
Yes	11 (40.7)	16 (59.3)	1.58 (0.53–4.72)
No	13 (52.0)	12 (48.0)	Reference
Presence of IVH, n (%)			
Yes	11 (32.4)	23 (67.6)	**5.44 (1.55–19.11)**
No	13 (72.2)	5 (27.8)	Reference
Hypertension, n (%)			
Yes	12 (52.2)	11 (47.8)	0.65 (0.22–1.95)
No	12 (41.4)	17 (58.6)	Reference
Active smoking, n (%)			
Yes	9 (45.0)	11 (55.0)	1.08 (0.35–3.31)
No	15 (46.9)	17 (53.1)	Reference
Anticoagulant and/or antiplatelet therapy, n (%)			
Yes	3 (33.3)	6 (66.7)	1.91 (0.42–8.64)
No	21 (48.8)	22 (51.2)	Reference
Timing of nimodipine from initial CT, n (%)			
Early (<7 days)	7 (38.9)	11 (61.1)	Reference
Late (≥7 days)	17 (50.0)	17 (50.0)	0.64 (0.20–2.03)
Angiographic improvement after IAN, n (%)			
Significant	6 (40.0)	9 (60.0)	1.42 (0.42–4.80)
No/mild	18 (48.6)	19 (51.4)	Reference
Repeated nimodipine, n (%)			
Yes	5 (21.7)	18 (78.3)	**6.84 (1.96–23.93)**
No	19 (66.5)	10 (34.5)	Reference
Angioplasty, n (%)			
Yes	0 (0.0)	4 (100.0)	
No	24 (50.0)	24 (50.0)	
Post-aneurysm treatment ischemic lesion, n (%)			
Yes	6 (42.9)	8 (57.1)	1.20 (0.35–4.13)
No	18 (47.4)	20 (52.6)	Reference
Overall ischemic lesion, n (%)			
Yes	8 (38.1)	13 (61.9)	1.73 (0.56–5.35)
No	16 (51.6)	15 (48.4)	Reference

Reference indicates reference category. Statistically significant comparisons in bold.

ABBREVIATIONS CI = confidence interval; DCI = delayed cerebral ischemia; GCS = Glasgow Coma Scale; IAN = intra-arterial nimodipine; ICH = intracerebral hemorrhage; IQR = interquartile range; IVH = intraventricular hemorrhage; mRS = Modified Rankin Scale, n = number of patients, OR = odds ratio.

In a multivariate model, older age was associated with increased six-month mortality, but not unfavorable outcomes (Table 3). In multivariate models, repeated IAN administration was not associated with six-month mortality (Table B). However, repeated IAN and the requirement of a cerebrospinal fluid shunt were associated with unfavorable functional outcomes at 3–6 months (Table B). Patients who required repeated IAN were more likely to require cerebrospinal fluid shunts (Table C). After propensity score matching for baseline severity of aSAH, repeated IAN was associated with unfavorable outcome compared to single IAN using Firth's penalized logistic regression (Table 4). Mortality did not differ significantly between single and repeated IAN infusion groups (Table 4).

**Table 3:** Outcomes of patients treated with intra-arterial nimodipine due to delayed cerebral ischemia after aneurysmal subarachnoid hemorrhage based on multivariate logistic regression. An unfavorable outcome was defined as mRS>2.

Characteristic	6-month mortality	Functional outcomes at 3–6 months
	Alive as the reference OR (95% CI)	Favorable as the reference OR (95% CI)
Age	**1.56 (1.04–1.30)**	1.01 (0.96–1.06)
Hunt and Hess grade	1.26 (0.14–11.11)	1.34 (0.45–3.99)
Fisher grade	N/A	3.81 (0.66–22.09)
Surgical treatment	1.03 (0.09–11.86)	1.27 (0.31–5.20)
GCS on admission	1.01 (0.63–1.64)	0.92 (0.69–1.22)

Statistically significant comparisons in bold.

N/A = not enough patients to form significant comparisons.

ABBREVIATIONS CI = confidence interval; GCS = Glasgow Coma Scale; mRS = Modified Rankin Scale; OR = odds ratio.

**Table 4:** Outcomes of patients treated with intra-arterial nimodipine using Firth's penalized logistic regression in the matched^a^ sample of 46 patients.

Characteristic	6-month mortality	Unfavorable outcome at 3–6 months
	OR (95% CI)	OR (95% CI)
Repeated nimodipine	0.74 (0.15–3.44)	**5.13 (1.53–19.35)**

An unfavorable outcome was defined as mRS>2.

ABBREVIATIONS CI = confidence interval; mRS = Modified Rankin Scale; OR = odds ratio.

a The propensity score matching included age, Hunt and Hess grade, Fisher grade, treatment modality and GCS on admission. Statistically significant comparisons in bold. Matched sample contained 23 patients with repeated nimodipine and 23 patients with single dosing.

## 4 Discussion

We examined functional outcomes and six-month mortality in patients with aSAH who received intra-arterial nimodipine (IAN) as rescue therapy for DCI. Fifty-four percent of the patients had unfavorable outcomes, consistent with the severity of their aSAH. As expected, high Hunt and Hess grade, high Fisher grade, low admission GCS and the presence of IVH were associated with poor outcomes. These factors reflect more extensive primary bleeding and impaired neurological status at ictus, which are well-established predictors of poor prognosis after aSAH (Starke et al., 2009; de Winkel et al., 2022). Similar to recent studies (Samuelsson et al., 2022; Vossen et al., 2023), the rate of favorable outcomes in the current study (46%) was considerably lower than that reported in early single-center IAN series, with rates of favorable outcome as high as 70–85% (Table 5). These differences likely reflect evolving patient selection, as older studies may have included less severely affected patients.

**Table 5:** Studies on patients treated with intra-arterial nimodipine for delayed cerebral ischemia after aneurysmal subarachnoid hemorrhage.

	n	CI	mRS 0–2/GOS>4 30d	mRS 0–2 (3–12m)/GOS 4–5	Mortality in hospital/30d	Mortality (3-12m)
Biondi et al., 2004^6^	25	76%	–	3–6m: 72%	–	3–6 m: 8%
Hui and Lau, 2005^19^	9	89%	–	–	0 (0%)	4m: 11%
Hänggi et al., 2008^7^	18	11%	61%	3m: 61%	1 (5%)	–
Kim et al., 2009^29^	19	68%	79%	–	0 (0%)	–
Cho et al., 2011^30^	42	68%	76%	6m: 85%	1 (2%)	–
Dehdashti et al., 2011^20^	10	60%	–	70%	–	20%
Kim et al., 2012^31^	29	72%	76%	–	2 (7%)	–
Bashir et al., 2016^1^	25	12%	4%	3m: 20%	9 (36%)	3m: 40%
Andereggen et al., 2017^21^	31	–	25%	10m: 60%	–	10m: 20%
Ditz et al., 2018^32^	15	–	0%	3/6m: 12%/36%	1 (7%)	–
Samuelsson et al., 2022^10^	48	–	25%	6m: 47%	2 (4%)/4 (8%)	6m: 13%
Vossen et al., 2024^18^	96	–	–	12m: 48%	–	12m: 30%
Our study	52	42%	–	3–6m: 46%	–	6m: 13%

CI = clinical improvement; d = days; GOS = Glasgow Outcome Scale; n = number of patients; m = months; mRS = modified Rankin Scale. – indicates no information. Modified from Samuelsson et al., 2022)^10^, studies on continuous IAN are excluded.

In our cohort, 3–6-month outcomes appeared more favorable in the single-dose IAN group (79%) compared to the repeated IAN group (52%). If IAN is beneficial, patients receiving repeat IAN would not be expected to have worse outcomes than the patients who receive IAN only once. However, repeat IAN was associated with worse functional outcome even after propensity score matching. In the HIMALAIA randomized trial, 12/20 (60%) patients with aSAH and secondary ischemia had favorable outcomes (mRs ≤ 3) at 3 months in the group that did not receive induced hypertension (Gathier et al., 2018). IAN was associated with the same infarction rate, but worse outcome compared to no IAN (Gathier et al., 2018). One possibility is that IAN may carry adverse effects that worsen outcome. A study comparing endovascular angioplasty combined with intravenous milrinone to only intensive therapy of DCI did not support the use of an interventional strategy over non-interventional treatment for improving outcomes (Labeyrie et al., 2021). The results after propensity score matching, suggest that any benefit of these therapies is likely to be small (Labeyrie et al., 2021). However, as sample sizes in studies are small, confounding rather than harmful effects of IAN are possible. Patients with poorer baseline status and more severe vasospasm more commonly required repeated IAN infusions (Kim et al., 2015; Vossen et al., 2023). A comparison of conservatively matched patients is needed in the future to answer this question.

The six-month mortality rate in our series (13%) was in line with most previous IAN studies (8– 20%) (Andereggen et al., 2017; Biondi et al., 2004; Dehdashti et al., 2011; Hui and Lau, 2005; Samuelsson et al., 2022), and slightly lower than in Finnish population-based series of all aSAH (18–20%) (Asikainen et al., 2023). A study of patients with DCI treated without intra-arterial medications reported a mortality rate of 24% (Gathier et al., 2018), although favorable functional outcomes in the current study (46%) were similar to outcomes in non-IAN DCI series (Gathier et al., 2018; Haegens et al., 2018; Tjerkstra et al., 2023). While IAN may not improve functional outcomes in post-aSAH DCI, there may be a trend toward reduced mortality (Table 6). However, due to baseline differences, our outcomes cannot be directly compared with patients who are managed conservatively only.

**Table 6:** Studies of patients treated without intra-arterial nimodipine for delayed cerebral ischemia after aneurysmal subarachnoid hemorrhage.

Studies on patients treated without IAN for DCI after aSAH.					
	n	mRS 0–2/GOS>4 30d	mRS 0–2 (3–12m)/GOS 4–5	Mortality in hospital/30d	Mortality (3-12m)
Haegens et al., 2018^24^	244	–	mRS 0–3 6m: 54%	–	–
Gathier et al., 2018^23^	41	–	3m: 39%	–	3m: 24%
Tjerkstra et al., 2023^25^, subgroup from UMCU^a^	76	–	mRS 0–3 6mo: 56%	12 (16%)	–
Our study	52	–	3–6m: 46%	–	6m: 13%

a Abbreviations: d = days; GOS = Glasgow Outcome Scale; n = number of patients; m = months; mRS = modified Rankin Scale. – indicates no information.Haegens et al., 2018. The subgroup that was the control group in the study was selected, because the other group included an institution that uses IAN.

The rate of post-IAN ischemic lesions was 15% in our series. Studies of non-responders to hypertensive therapy have found higher infarction rates (20–44%) when IAN was not administered (Suwatcharangkoon et al., 2019). This observation raises the possibility that IAN may reduce infarction formation in patients who fail to respond to induced hypertension. However, in another IAN study, infarcts were observed in 60% of patients (Samuelsson et al., 2022). While the retrospective setting of our study precludes causal inference, the comparatively low infarction rate may suggest a role for nimodipine in limiting ischemic damage. This could represent a clinically meaningful benefit, although confirmation in randomized trials is required.

Angiographic improvement in vasospasm following IAN was minimal in most patients. Although only 29% of patients showed substantial improvement of spasmic vessels angiographically, GCS scores improved in 42% after IAN. The benefits of IAN are typically observed within few hours as clinical improvements in the symptoms of vasospasm. Consistent with our findings, 76% of 25 patients had substantial clinical improvement within 24 h following IAN, although angiographic response (≥30% diameter increase) was poor in 57% of cases in another study (Biondi et al., 2004).

Older age and pre-ictus anticoagulant or antiplatelet therapy were associated with higher mortality in our study. This is consistent with prior literature and likely reflects larger initial bleeds, and higher baseline cardiovascular morbidity with poorer overall physical condition in these patients (Hao et al., 2022; Kultanen et al., 2023). Rather than a direct pharmacologic interaction with nimodipine, these associations probably arise from more fragile baseline cerebrovascular physiology, higher comorbidity burden, and reduced physical and recovery capacity.

Despite the relatively modest cohort size (n = 52), our study represents one of the larger series examining IAN in the treatment of cerebral vasospasm, highlighting the overall scarcity of robust clinical data in this field. This is underscored by the only randomized controlled trial to date, the IMCVS trial, which included 34 patients and compared invasive endovascular therapy with conservative management using induced hypertension (Vatter et al., 2022). Notably, IMCVS demonstrated no reduction in DCI with invasive treatment and reported significantly worse functional outcomes at 6 months in the endovascular group (Vatter et al., 2022). Taken together, both the randomized IMCVS data and our comparatively larger real-world cohort support a more selective, indication-driven use of these interventions in this challenging and under-investigated area.

Our findings suggest that patients receiving IAN represent a severely affected subgroup of aSAH patients with poor baseline prognosis. Functional outcomes were not superior to those in non-IAN series, but mortality appeared slightly lower. Taken together, IAN may have a role in preventing cerebral infarction and possibly reducing mortality, although overall functional recovery benefit may be limited. Prospective randomized studies would be required to establish whether IAN provides true benefit beyond current intensive care and induced hypertension strategies.

## 5 Limitations

This study has several limitations. Most importantly, the retrospective design lacks randomization, limiting the comparability of outcomes between patients who did and did not receive IAN. Variation in outcome assessment timing (3–6 months) may influence outcome differences. In addition, the relatively small sample size and single-center setting reduce the generalizability of the findings, as treatment protocols, diagnostic criteria, and dosing strategies vary considerably across institutions (Ditz et al., 2018; Samuelsson et al., 2022). Although propensity score matching was used to balance baseline characteristics between the repeated and single-dose IAN groups, H&H grade and GCS on admission remained imbalanced after matching, indicating that residual confounding by indication may partly explain the unfavorable outcomes observed with repeated IAN. However, the strict and unconditional protocol for IAN administration at our institution strengthens the reliability of the results despite the retrospective nature of the study, as treatment was applied consistently to all patients unresponsive to fluid optimization and induced hypertension.

## 6 Conclusions

In this cohort of aSAH patients with vasospasm-related DCI treated with IAN, repeated IAN was associated with unfavorable outcomes. However, mortality did not differ significantly between single and repeated IAN groups. Earlier treatment was not associated with improved outcomes.

## Authors contributed

All authors contributed to the writing of the manuscript at the draft and revision stages and have read and approved the final version.

Experimental design: T.K.K., J.-M.I., M.F., C.Q.

Implementation: S.P., J.-M.I., M.F., C.Q.

Analysis and interpretation of the data: E.K.P., S.P., T.K.K., J.-M.I., C.Q.

## Data statement

Data statement: Anonymized data will be made available upon reasonable request to the corresponding author (subject to the Finnish regulations).

## Declaration of generative AI and AI-assisted technologies in the manuscript preparation process

Statement: During the preparation of this work the authors used ChatGPT in order to check the grammar and readability of the work. After using this tool/service, the authors reviewed and edited the content as needed and take full responsibility for the content of the published article.

## Funding

No funding was received for this research.

## Declaration of competing interest

The authors declare that they have no known competing financial interests or personal relationships that could have appeared to influence the work reported in this paper.
