# Supplementary material for: Intra-arterial nimodipine and outcomes in delayed cerebral ischemia after aneurysmal subarachnoid hemorrhage
Source: Brain Spine. 2026 Aug 10;6:106281. doi: 10.1016/j.bas.2026.106281 (PMC13507813; doi:10.1016/j.bas.2026.106281)
Supplement: Supplementary file 1 [file mmc1.docx]

**Table A.** Outcomes of patients treated with intra-arterial nimodipine due to delayed cerebral ischemia after aneurysmal subarachnoid hemorrhage based on univariate logistic regression. Seven patients died and 45 were alive (reference category).

| Characteristic | 6-month mortality | |
| --- | --- | --- |
|  | OR (95% CI) |  |
| Mean age | **1.13 (1.02–1.25)** |  |
| Sex |  |  |
| Female | Reference |  |
| Male | 2.95 (0.58–14.99) |  |
| Hunt and Hess grade |  |  |
| 1–3 | Reference |  |
| 4–5 | 2.42 (0.48–12.17) |  |
| Fisher grade | N/A |  |
| Aneurysm location |  |  |
| Anterior circulation | Reference |  |
| Posterior circulation | 0.91 (0.09–8.72) |  |
| Treatment |  |  |
| Surgery | 0.80 (0.14–4.62) |  |
| Endovascular | Reference |  |
| GCS on admission |  |  |
| 13–15 | Reference |  |
| 9–12 | 2.58 (0.21–31.20) |  |
| 3–8 | 3.10 (0.54–17.87) |  |
| Presence of ICH |  |  |
| Yes | 0.12 (0.01–1.10) |  |
| No | Reference |  |
| Presence of IVH |  |  |
| Yes | 3.64 (0.40–32.91) |  |
| No | Reference |  |
| Hypertension |  |  |
| Yes | 1.83 (0.37–9.12) |  |
| No | Reference |  |
| Active smoking |  |  |
| Yes | 0.60 (0.11–3.44) |  |
| No | Reference |  |
| Anticoagulant and/or antiplatelet therapy |  |  |
| Yes | **10.67 (1.83–62.13)** |  |
| No | Reference |  |
| Timing of nimodipine from initial CT |  |  |
| Early (<7 days) | Reference |  |
| Late (≥7 days) | 0.67 (0.13–3.37) |  |
| Repeated nimodipine |  |  |
| Yes | 0.94 (0.18–4.68) |  |
| No | Reference |  |
| Angioplasty |  |  |
| Yes | 1.87 (0.29–12.33) |  |
| No | Reference |  |
| Post-aneurysm treatment ischemic lesion |  |  |
| Yes | 0.41 (0.05–3.75) |  |
| No | Reference |  |
| Post-IAN treatment ischemic lesion |  |  |
| Yes | 2.60 (0.41–16.56) |  |
| No | Reference |  |
| Overall ischemic lesion |  |  |
| Yes | 0.55 (0.96–3.13) |  |
| No | Reference |  |
| Reference indicates reference category. Statistically significant comparisons in bold.  **ABBREVIATIONS** CI = confidence interval; GCS = Glasgow Coma Scale; ICH = intracerebral hemorrhage; IVH = intraventricular hemorrhage; OR = odds ratio. | | |

**Table B.** Outcomes of patients treated with intra-arterial nimodipine due to delayed cerebral ischemia after aneurysmal subarachnoid hemorrhage based on multivariate logistic regression. An unfavorable outcome was defined as mRS>2.

| Characteristic | 6-month mortality | | | | | Unfavorable outcome at 3–6 months | | |
| --- | --- | --- | --- | --- | --- | --- | --- | --- |
|  | OR (95% CI) | | | |  | OR (95% CI) | | |
| Timing of nimodipine from initial CT | |  |  |  |  |  |  |  |
| Early (<7 days) | Reference | | | |  | Reference | | |
| Late (≥7 days) | 1.51 (0.21–11.04) | | | |  | 1.21 (0.29–5.09) | | |
| Repeated nimodipine | 1.32 (0.15–11.54) | | | |  | **6.39 (1.52–26.81)** | | |
| EVD | N/A | | | |  | 6.82 (0.62–75.39) | | |
| Cerebrospinal fluid shunt | 0.64 (0.04–10.22) | | | |  | **8.18 (1.33–50.48)** | | |
| Number of vessels with significant spams, n (%) |  | | | |  |  | | |
| 1 | Reference | | | |  | Reference | | |
| 2 | 0.44 (0.04–5.14) | | | |  | 1.63 (0.38–6.95) | | |
| ≥3 | 0.48 (0.04–6.39) | | | |  | 1.66 (0.26–10.72) | | |
| Angiographic improvement after IAN, n (%) |  | | | |  |  | | |
| Significant | 0.32 (0.04–2.70) | | | |  | 0.42 (0.09–1.98) | | |
| No/mild | Reference | | | |  | Reference | | |

The models were adjusted with age, Hunt and Hess grade, Fisher grade, treatment modality and GCS on admission. Statistically significant comparisons in bold.

**ABBREVIATIONS** CI = confidence interval; GCS = Glasgow Coma Scale; EVD = external ventricular drain; mRS = Modified Rankin Scale; OR = odds ratio.

**Table C.** Characteristics of patients grouped by repeated intra-arterial nimodipine infusion.

| Characteristic | Repeated nimodipine | | |  |
| --- | --- | --- | --- | --- |
|  | Yes  (n = 23) | No  (n = 29) (Reference) | OR (95% CI) |  |
| Mean age (SD) | 50 (11) | 55 (14) | 0.97 (0.93–1.02) |  |
| Sex n (%) |  |  |  |  |
| Female | 17 (50.0) | 17 (50.0) | Reference |  |
| Male | 6 (33.3) | 12 (66.7) | 0.50 (0.52–1.64) |  |
| Hunt and Hess grade, n (%) |  |  |  |  |
| 1–3 | 12 (37.5) | 20 (62.5) | Reference |  |
| 4–5 | 11 (55.0) | 9 (45.0) | 2.04 (0.66–6.34) |  |
| Fisher grade, n (%) |  |  |  |  |
| 0–3 | 3 (27.3) | 8 (72.7) | Reference |  |
| 4 | 20 (48.8) | 21 (51.2) | 2.54 (0.59–10.95) |  |
| Aneurysm location, n (%) |  |  |  |  |
| Anterior circulation | 18 (40.9) | 26 (59.1) | Reference |  |
| Posterior circulation | 5 (62.5) | 3 (37.5) | 2.41 (0.51–11.37) |  |
| Treatment, n (%) |  |  |  |  |
| Surgery | 8 (47.1) | 9 (52.9) | 1.19 (0.37–3.80) |  |
| Endovascular | 15 (42.9) | 20 (57.1) | Reference |  |
| Mean GCS on admission (SD) | 10.2 (5.1) | 12.5 (4.0) | 0.89 (0.79–1.01) |  |
| Presence of ICH, n (%) |  |  |  |  |
| Yes | 14 (51.9) | 13 (48.1) | 1.92 (0.63–5.82) |  |
| No | 9 (36.0) | 16 (64.0) | Reference |  |
| Presence of IVH, n (%) |  |  |  |  |
| Yes | 18 (52.9) | 16 (47.1) | 2.93 (0.85–10.03) |  |
| No | 5 (27.8) | 13 (72.2) | Reference |  |
| Hypertension, n (%) |  |  |  |  |
| Yes | 9 (39.1) | 14 (60.9) | 0.67 (0.23–2.09) |  |
| No | 14 (60.9) | 25 (51.7) | Reference |  |
| Active smoking, n (%) |  |  |  |  |
| Yes | 10 (50.0) | 10 (50.0) | 1.46 (0.47–4.50) |  |
| No | 13 (40.6) | 19 (59.4) | Reference |  |
| Anticoagulant and/or antiplatelet therapy, n (%) |  |  |  |  |
| Yes | 3 (33.3) | 6 (66.7) | 0.58 (0.13–2.60) |  |
| No | 20 (46.5) | 23 (53.5) | Reference |  |
| Timing of nimodipine from initial CT, n (%) |  |  |  |  |
| Early (<7 days) | 8 (44.4) | 10 (55.6) | Reference |  |
| Late (≥7 days) | 15 (44.1) | 19 (55.9) | 0.99 (0.31–3.12) |  |
| Post-aneurysm treatment ischemic lesion, n (%) |  |  |  |  |
| Yes | 5 (35.7) | 9 (64.3) | 0.62 (0.17–2.19) |  |
| No | 18 (47.4) | 20 (52.6) | Reference |  |
| Post-IAN treatment ischemic lesion, n (%) |  |  |  |  |
| Yes | 5 (62.5) | 3 (37.5) | 2.41 (0.51–11.37) |  |
| No | 18 (40.9) | 26 (59.1) | Reference |  |
| Overall ischemic lesion, n (%) |  |  |  |  |
| Yes | 10 (47.6) | 11 (52.4) | 1.26 (0.41–3.84) |  |
| No | 13 (41.9) | 18 (58.1) | Reference |  |
| 6-month mortality, n (%) |  |  |  |  |
| Dead | 3 (42.9) | 4 (57.1) | 0.94 (0.19–4.68) |  |
| Alive | 20 (44.4) | 25 (55.6) | Reference |  |
| Outcome according to mRS |  |  |  |  |
| Favorable (0–2) | 5 (20.8) | 19 (79.2) | Reference |  |
| Unfavorable (3–6) | 18 (64.3) | 10 (35.7) | **6.84 (1.96–23.93)** |  |
| Increase of GCS after IAN admission, n (%) |  |  |  |  |
| Yes | 11 (50.0) | 11 (50.0) | 0.67 (0.22–2.02) |  |
| No | 12 (40.0) | 18 (60.0) | Reference |  |
| Permanent shunt, n (%) |  |  |  |  |
| Yes | 11 (73.3) | 4 (26.7) | **5.73 (1.51–21.78)** |  |
| No | 12 (32.4) | 25 (67.6) | Reference |  |

Statistically significant comparisons in bold.

**ABBREVIATIONS** CI = confidence interval; GCS = Glasgow Coma Scale; IAN = intra-arterial nimodipine; ICH = intracerebral hemorrhage; IVH = intraventricular hemorrhage; mRS = Modified Rankin Scale, n = number of patients; OR = odds ratio SD = standard deviation.

**Table D.** Characteristics of patients grouped by nimodipine infusion timing.

| Characteristic | Timing of nimodipine from initial CT | | | |
| --- | --- | --- | --- | --- |
|  | Early (<7 days) (n = 18) (Reference) | Late (≥7 days) (n=34) | OR (95% CI) |  |
| Mean age (SD) | 53 (9) | 51 (14) | 0.96 (0.91–1.01) |  |
| Sex n (%) |  |  |  |  |
| Female | 9 (26.5) | 25 (73.5) | Reference |  |
| Male | 9 (50.0) | 9 (50.0) | 0.36 (0.11–1.19) |  |
| Hunt and Hess grade, n (%) |  |  |  |  |
| 1–3 | 9 (28.1) | 23 (71.9) | Reference |  |
| 4–5 | 9 (45.0) | 11 (55.0) | 0.48 (0.15–1.54) |  |
| Mean Fisher grade (SD) | 3.9 (0.3) | 3.7 (0.5) | 0.35 (0.07–1.71) |  |
| Aneurysm location, n (%) |  |  |  |  |
| Anterior circulation | 15 (34.1) | 29 (65.9) | Reference |  |
| Posterior circulation | 3 (37.5) | 5 (62.5) | 0.86 (0.18–4.11) |  |
| Treatment, n (%) |  |  |  |  |
| Surgery | 8 (47.1) | 9 (52.9) | Reference |  |
| Endovascular | 10 (28.6) | 25 (71.4) | 0.45 (0.14–1.50) |  |
| Mean GCS on admission (SD) | 9.9 (5.1) | 12.4 (4.2) | 1.12 (0.99–1.27) |  |
| Presence of ICH, n (%) |  |  |  |  |
| Yes | 13 (48.1) | 14 (51.9) | Reference |  |
| No | 5 (20.0) | 20 (80.0) | **0.27 (0.08–0.93)** |  |
| Presence of IVH, n (%) |  |  |  |  |
| Yes | 11 (32.4) | 23 (67.6) | Reference |  |
| No | 7 (38.9) | 11 (61.1) | 1.33 (0.41–4.37) |  |
| Hypertension, n (%) |  |  |  |  |
| Yes | 8 (34.8) | 15 (65.2) | Reference |  |
| No | 10 (34.5) | 19 (65.5) | 0.99 (0.31–3.12) |  |
| Active smoking, n (%) |  |  |  |  |
| Yes | 10 (50.0) | 10 (50.0) | Reference |  |
| No | 8 (25.0) | 24 (75.0) | 0.33 (0.10–1.09) |  |
| Post-aneurysm treatment ischemic lesion, n (%) |  |  |  |  |
| Yes | 4 (28.6) | 10 (71.4) | Reference |  |
| No | 14 (36.8) | 24 (63.2) | 1.46 (0.38–5.54) |  |
| Post-IAN treatment ischemic lesion, n (%) |  |  |  |  |
| Yes | 6 (75.0) | 2 (25.0) | Reference |  |
| No | 12 (27.3) | 32 (72.7) | **0.13 (0.02–0.71)** |  |
| Overall ischemic lesion, n (%) |  |  |  |  |
| Yes | 9 (42.9) | 12 (57.1) | Reference |  |
| No | 9 (29.0) | 22 (71.0) | 0.55 (0.17–1.74) |  |
| Permanent shunt, n (%) |  |  |  |  |
| Yes | 4 (26.7) | 11 (73.3) | Reference |  |
| No | 14 (37.8) | 23 (62.2) | 1.67 (0.45–6.29) |  |

Statistically significant comparisons in bold.

**ABBREVIATIONS** CI = confidence interval; GCS = Glasgow Coma Scale; IAN = intra-arterial nimodipine; ICH = intracerebral hemorrhage; IVH = intraventricular hemorrhage; mRS = Modified Rankin Scale; n = number of patients, OR = odds ratio; SD = standard deviation.

**Figure A.** LOVE plot for the propensity score matching analysis.

Absolute Mean Differences

Note: The LOVE plot displays the standardized difference in baseline characteristics between the repeated and single-dose IAN groups, before (unmatched) and after (matched) propensity score matching. Values closer to zero indicate better balance between groups. Matching improved balance for most characteristics; however, H&H grade and GCS on admission remained relatively imbalanced even after matching, suggesting a degree of residual confounding by indication that should be considered when interpreting the matched analysis (see Limitations).
